# *HLA-DR* and *HLA-DQ* Polymorphism Correlation with Sexually Transmitted Infection Caused by *Chlamydia trachomatis*

**DOI:** 10.3390/medicina60050808

**Published:** 2024-05-14

**Authors:** Martina-Luciana Pintea-Trifu, Mihaela Laura Vică, Silvia-Ștefana Bâlici, Daniel-Corneliu Leucuța, Horia George Coman, Bogdan Nemeș, Dragoș-Mihail Trifu, Costel-Vasile Siserman, Horea-Vladi Matei

**Affiliations:** 1 Department of Cellular and Molecular Biology, Iuliu Hațieganu University of Medicine and Pharmacy, 400349 Cluj-Napoca, Romania; pintea.trifu.martina@elearn.umfcluj.ro (M.-L.P.-T.); sbalici@umfcluj.ro (S.-Ș.B.); hmatei@umfcluj.ro (H.-V.M.); 2 Department of Medical Informatics and Biostatistics, Iuliu Hațieganu University of Medicine and Pharmacy, 400349 Cluj-Napoca, Romania; 3 Department of Medical Psychology and Psychiatry, Iuliu Hațieganu University of Medicine and Pharmacy, 400012 Cluj-Napoca, Romania; hcoman@umfcluj.ro (H.G.C.); nemes.bogdan@umfcluj.ro (B.N.); 4 Department of Urology, Regina Maria Cluj Hospital, 400696 Cluj-Napoca, Romania; trifu.dragos.mihail@elearn.umfcluj.ro; 5 Department of Forensic Medicine, Iuliu Hațieganu University of Medicine and Pharmacy, 400006 Cluj-Napoca, Romania; cvsiserman@gmail.com

**Keywords:** sexually transmitted diseases, *Chlamydia trachomatis*, MHC class II, *HLA* antigens, *HLA-DRB1*, *HLA-DQB1*

## Abstract

*Background and Objectives*: *Chlamydia trachomatis (C. trachomatis)* represents one of the most prevalent bacterial sexually transmitted diseases. This study aims to explore the relationship between HLA alleles/genotypes/haplotypes and *C. trachomatis* infection to better understand high-risk individuals and potential complications. *Materials and Methods*: This prospective study recruited participants from Transylvania, Romania. Patients with positive NAAT tests for *C. trachomatis* from cervical/urethral secretion or urine were compared with controls regarding *HLA-DR* and *-DQ* alleles. DNA extraction for HLA typing was performed using venous blood samples. *Results*: Our analysis revealed that the presence of the DRB1*13 allele significantly heightened the likelihood of *C. trachomatis* infection (*p* = 0.017). Additionally, we observed that individuals carrying the DRB1*01/DRB1*13 and DQB1*03/DQB1*06 genotype had increased odds of *C. trachomatis* infection. Upon adjustment, the association between the DRB1*01/DRB1*13 genotype and *C. trachomatis* remained statistically significant. *Conclusions*: Our findings underscore the importance of specific *HLA* alleles and genotypes in influencing susceptibility to *C. trachomatis* infection. These results highlight the intricate relationship between host genetics and disease susceptibility, offering valuable insights for targeted prevention efforts and personalized healthcare strategies.

## 1. Introduction

The global health sector strategies for sexually transmitted infections (STIs) during the period 2022–2030 aim to reduce the number of new cases of chlamydia, syphilis, gonorrhea, and trichomoniasis to less than 150 million by 2030 [1]. Estimated incident cases in 2016 totaled 376.4 million, including 127.2 million cases of chlamydia, 86.9 million cases of gonorrhea, 156.0 million cases of trichomoniasis, and 6.3 million cases of syphilis [2]. *Chlamydia trachomatis* (*C. trachomatis*) represents one of the most prevalent sexually transmitted bacteria. Although chlamydia notification rates peaked in 2019, there was a decrease in notifications during 2020 and 2021 amid the COVID-19 pandemic. However, new record-high notification rates were observed in both women and men for the year 2022 [3]. A critical component of the global health sector’s strategy is to promote accelerated research and development of prevention technologies, diagnostics, therapy, and vaccines for STIs [1]. 

Infection is influenced by the biggest number of genes expressed with diversity, showing how the human body has evolved immunologically through a great number of pathogens [4,5]. The human leukocyte antigen (HLA) system plays an essential role in the regulation of the immune function, determining the self from non-self, and thus taking part in solid organ transplantation, hematopoietic stem cell transplantation, transfusion medicine, and the association of disease with pharmacogenomic applications. The HLA genes are localized in the most polymorphic region of the human genome, the region of the major histocompatibility complex (MHC), on the 6p21.3 chromosome. There are over 20000 closely related but distinct alleles in the MHC system coding class l loci (*HLA-A, HLA-B, HLA-C, HLA-E, HLA-F, HLA-G*) and class ll loci (*HLA-DR*, *HLA-DQ*, *HLA-DM, HLA-DP*) [6]. Lying between classes l and ll, some other genes that regulate innate immunity have been called MHC class III (complement C2 and C4B, tumor necrosis factor-gene TNF, lymphotoxin-alfa-gene LTA, genes like HSPA 1A and 1B, LTB, MICA, MICB) [7]. HLA from parents is inherited as a haplotype, or sets of genes on contiguous loci. Haplotypes are Mendelian inherited [8,9]. 

In the context of the association between HLA and the onset of specific diseases, it is recognized that immune mechanisms play a role alongside genetic predisposition attributed to HLA factors and other genes. Currently, two explanations have been proposed. Firstly, there may exist an imbalance between alleles from the locus linked to a disease and the HLA allele correlated with that disease (such as HLA-A3 and idiopathic hemochromatosis). The second explanation suggests that HLA antigens contribute to the disease process by inadequately presenting bacterial or viral antigens; providing binding sites on the cell surface for pathogenic microorganisms; serving as a transport mechanism for viruses, facilitating their entry into cells; and mimicking the structure of pathogenic molecules and deceiving the immune system into not recognizing them as foreign. These mechanisms may act in concert to predispose individuals to certain diseases. Conversely, HLA polymorphism could also exert a protective effect against disease development, as observed with HLA-DQB1*0602, which confers nearly complete protection against insulin-dependent diabetes mellitus [10].

In a comprehensive literature search, several HLA alleles influencing susceptibility to infection/reinfection/appearance of complications of *C. trachomatis* infection were found. Most of these were among the *HLA-DR1* and *HLA-DQ1* genes [11,12].

A review of the medical literature reveals the identification of HLA alleles that confer resistance or protection against *C. trachomatis* infection. However, there is a notable gap in studies examining HLA alleles within European populations, including the Romanian population. Existing studies report associations with various alleles, yet consistency across the literature is lacking. Notably, certain HLA alleles have been predominantly associated with Afro-American populations. Among these, statistically significant associations were most frequently observed with *HLA-DR* and *HLA-DQ.*

The HLA alleles associated with susceptibility to *C. trachomatis* infection include A*23 [13], A*28 (linked to trachoma) [14], A*31 (associated with pelvic inflammatory disease) [15], A*36 (related to reinfection) [16], B*08 [13], B*14 (associated with trachoma) [17], DRB1*01, DRB1*03, DRB1*08, DRB1*11 (linked to persistence), DRB1*13 (linked to persistence), DRB1*14 [18], DRB1*15 [16], DRB1*16 [18], DQA*01 [19], DQA*03, DQA*05, DQB*02 [20], DQB*05 (associated with infertility) [21], DQB*06 (related to reinfection) [20], DQB1*05 (associated with persistence), and DQB1*06 (associated with reinfection) [16]. Other alleles providing protection or resistance against infection include B*08 [13], Cw*16 [16], DRB1*04 [18], DRB1*09 [18], DRB1*10 [18], DRB1*11 [18], DRB1*12 [18], DQA*01 [19], and DQB1*05 [19]. Studies on macaques have indicated that *HLA-A*, *-B*, and -*C* alleles are associated with the early formation of adhesions [22].

The genes *HLA-DRB3/4/5* are closely associated with the *HLA-DRB1* locus [23]. While the expression levels of *HLA-DRB3*, *HLA-DRB4*, and *HLA-DRB5* are lower compared with those of *HLA-DRB1*, they remain detectable through serological methods, leading to their designation as *HLA-DR52*, *-DR53*, and *-DR51* antigens, respectively [24]. Additionally, non-expressed alleles are prevalent within the *HLA-DRB3/4/5* genes, with HLA-DRB4*01:03:01:02N being the most common HLA-null allele, observed with an overall frequency of approximately 3.5% [25,26]. *HLA-DRB3/4/5* have roles in organ transplant matching but were also found correlated to certain STIs, such as chronic HCV infection, where *HLA DR3* was associated with cryoglobulinemia [27].

The aim of this study was to investigate the relation between HLA-DR and HLA-DQ alleles/genotypes/haplotypes and *C. trachomatis* infection, in order to enhance our comprehension of individuals at elevated risk of acquiring the disease, experiencing complications, or developing severe symptoms. This knowledge can inform vaccine design to target specific populations more effectively and contribute to the advancement of personalized healthcare approaches.

## 2. Materials and Methods

### 2.1. Study Design and Location

This prospective investigation was conducted within Transylvania, a geographical region of Romania, which includes the following counties: Alba, Arad, Bihor, Bistrița-Năsăud, Brașov, Cluj, Covasna, Harghita, Hunedoara, Maramureș, Mureș, Satu-Mare, Sălaj, and Sibiu [28]. Participants were recruited from various public and private hospitals in the counties of Cluj, Bistrița-Năsăud, and Sălaj, where subjects from the entire region assembled to undergo genetic testing. The healthcare institutions included were “Iuliu Hațieganu” University of Medicine and Pharmacy Cluj-Napoca, County Emergency Hospital Cluj-Napoca, County Emergency Hospital Bistrița, Sanovil Clinic Bistrița, Bistrița Sanitary Theoretical High-School (Post Secondary School), The Neuropsychiatric Recovery and Rehabilitation Center for Youth in Beclean, and “Prof. Dr. Ioan Pușcaș” City Hospital Șimleu Silvaniei. Consecutive sampling was employed for recruitment within dermatology, obstetrics-gynecology, and urology departments, while convenience sampling was utilized for other patient sources. Data collection spanned from November 2021 to February 2024. The study comprised two groups of individuals: the *C. trachomatis* positive group and the control group (healthy subjects).

#### Informed Consent and Ethical Approval

For the acquisition of data and biological specimens (urine, vaginal or urethral secretion, and blood) participants in the study were required to provide informed consent, which explicitly outlined the collection of information via questionnaires and samples. All documentation and procedural protocols obtained approval from the Ethics Committee of the “Iuliu Hațieganu” University of Medicine and Pharmacy in Cluj-Napoca (notice number AVZ10 dated 8 November 2021) as well as the respective ethics committees of the public and private institutions where the study was conducted: Bistrița Sanitary Theoretical High-School (Postsecondary School)—notice number 2531 dated 15 November 2021, Neuropsychiatric Recovery and Rehabilitation Center for Youth in Beclean—notice number 4939 dated 22 November 2021, “Prof. Dr. Ioan Pușcaș” City Hospital Șimleu Silvaniei—notice number 3521 from 21 December 2021, County Emergency Hospital Cluj-Napoca—notice number 4980 dated 10 February 2022. All personal data pertaining to enrolled subjects and this data’s handling adhere to EU Regulation no. 679/2016 (concerning the protection of individuals regarding the processing of personal data and the free movement of such data), a regulation that became effective in Romania on 25 May 2018. Patient information, managed in accordance with GDPR regulations, encompassed a wide range of aspects, including general patient data, demographic information, income source, sexual history, environmental context, STI history, symptomatology, comorbidities, sexual orientation, and the presence of partners with STIs.

### 2.2. Participants

We endeavored to identify individuals afflicted with *C. trachomatis* infection through nucleic acid amplification testing (NAAT), employing Real-time Polymerase Chain Reaction (RT-PCR) for molecular diagnosis. We underwent urine/vaginal/urethral secretion testing for adults (aged 18 years and above) seeking medical appointments due to symptoms indicative of a bacterial STI (such as *C. trachomatis*, *Neisseria gonorrhoeae*, *Mycoplasma genitalium*, and similar pathogens); individuals identified as sexual partners of patients infected with a sexually transmitted pathogen, who were born in the region of Transylvania, Romania (comprising the counties of Alba, Arad, Bihor, Bistrița-Năsăud, Brașov, Cluj, Covasna, Harghita, Hunedoara, Maramureș, Mureș, Satu-Mare, Sălaj, and Sibiu); and those who provided informed consent for study involvement and genetic testing. Our case group exclusively comprised patients with a confirmed molecular diagnosis of *C. trachomatis*, out of all the patients tested. Evaluation of sexual partners of infected patients was conducted using a questionnaire. Exclusion criteria encompassed the absence of *C. trachomatis* in the NAAT test and refusal to participate in the study. For the control group, inclusion criteria involved healthy adults (aged 18 years and above) who were not hospitalized, and were born in the same geographical region as the subjects from the study group (the same Transylvanian counties of Romania: Alba, Arad, Bihor, Bistrița-Năsăud, Brașov, Cluj, Covasna, Harghita, Hunedoara, Maramureș, Mureș, Satu-Mare, Sălaj, and Sibiu), were unrelated, were from the same age group as the patients in the study group, and who shared similar environmental characteristics and agreed to undergo genetic testing, chosen in a consecutive sampling manner. Exclusion criteria for the control group included hospitalization at the time of enrollment, birthplace other than the above-mentioned geographical region, having relatives (related by blood) included in the control group, and those who refused genetic testing.

### 2.3. Variables

The data collection process involved administering questionnaires to participants under the supervision of the investigator, and biological sample collection of first void urine samples collected in the morning; vaginal or urethral secretion—for the detection of *C. trachomatis* presence/absence through RT-PCR; and 2 mL of peripheral venous blood collected in anticoagulant ethylenediaminetetraacetic acid (EDTA)—for genomic DNA extraction for *HLA-DR* and *HLA-DQ* determination. The questionnaires predominantly comprised multiple-choice questions. For age, numerical responses were required. Response options for gender included female, male, and other. 

#### 2.3.1. DNA Extraction from Cervical/Urethral Swabs and Urine Sediment and Testing for *C. trachomatis*

The biological samples were collected in medical facilities equipped in accordance with current laws and regulations, and they were transported and stored following the manufacturer’s instructions for the extraction kit used and for cervical and urethral swabs and urine sediment specimens. The kit used for DNA extraction and PCR amplification was the *N. gonorrhoeae*/*C. trachomatis*/*M. genitalium* Real-TM kit (Sacace Biotechnologies SRL Company, 22100 Como, Italy). 

The RT-PCR program used in Applied Biosystems^®^ 7500 Real-Time PCR (Applera) is shown in Table 1.

The concentration and purity of the DNA were assessed utilizing a Pearl nanophotometer (Implen GmbH, Munich, Germany). DNA samples with an A280/A260 ratio falling within the range of 1.8 ± 10% were deemed suitable for amplification. Samples not meeting this criterion underwent purification using the EPICENTRE MasterPureTM Complete DNA and RNA Purification Kit (Illumina Company, Madison, WI, USA). Interpretation of results relied on the presence of a fluorescence curve crossing the threshold line.

#### 2.3.2. DNA Extraction from Blood and HLA Typing

For *HLA-DR* and -*DQ* typing, DNA was extracted from blood using the Inno Train Ready DNA Isolation Spin Kit (Inno Train Diagnostik GmbH Company, D-61476 Kronberg/Taunus, Germany). Samples with an A280/A260 ratio of 1.8 ± 10% were considered appropriate for typing.

Typing of *HLA-DRB1* and *HLA-DQB1*, belonging to the MHC HLA class II, was performed utilizing the sequence-specific priming polymerase chain reaction (SSP-PCR) technique, employing the HLA-FluoGene *DRDQ* kit (Inno Train Diagnostik GmbH Company, D-61476 Kronberg/Taunus, Germany). DNA amplification was carried out using a G-Storm thermal cycler (Gene Technologies Ltd., Essex, UK). The fluorescence signals of the PCR products were detected using the FluoVista Analyzer (Inno Train Diagnostik GmbH Company, D-61476 Kronberg/Taunus, Germany). The endpoint fluorescence of the various fluorochromes before and after PCR was automatically calculated via the FluoGene analysis software (Inno Train Diagnostik GmbH Company, D-61476 Kronberg/Taunus, Germany) [29].

#### 2.3.3. Statistical Analysis

Counts and percentages were used to describe categorical variables. Medians and interquartile ranges were used to describe data that did not follow the normal distribution. Either the chi-squared test or the Fisher exact test (in case of low expected frequencies) was used to assess the relationship between two categorical variables. The Wilcoxon rank-sum test was used to compare two independent groups regarding data not following the normal distribution. Two multiple logistic regression models were built, with *C. trachomatis* as the dependent variable and a genotype as the independent variable of interest, along with two variables used for adjustment for age and sex. Initially the models were fit with the variable age as a continuous one, but we found that it had a non-linear association with the logit of the model. We checked this using a generalized additive model with splines. Therefore, we dichotomized the age variable by the median, and then we refit the models. Furthermore, we assessed the models’ goodness-of-fit with the Hosmer and Lemeshow test, and we checked the assumption of multicollinearity with variance inflation factors. The models were presented by odds ratios, 95% confidence intervals, *p*-values, and the area under the receiver operating characteristic curve. A value of 0.05 was used as the level of statistical significance. The frequencies of haplotypes were calculated for the *C. trachomatis* and control groups. In order to evaluate the link between HLA haplotypes and inclusion in the *C. trachomatis* group, we employed scoring tests [30]. The haplotype analyses were performed using the haplo.stats R package version 1.9.3 [31]. All analyses were carried out within the R environment for statistical computing and graphics (R Foundation for Statistical Computing, Vienna, Austria), version 4.3.3 [32].

## 3. Results

We tested 307 patients with bacterial STI symptoms and sexual partners of patients infected with *C. trachomatis*. Out of the 307 persons who underwent NAAT testing through RT-PCR, 12.37% (*n* = 38) were positive for chlamydia. Only the patients with a molecular diagnosis of *C. trachomatis* were included in the study group. Participants in this study were divided into two groups: the *C. trachomatis* group (*n* = 38) and the control group (*n* = 467). The median age of participants in the *C. trachomatis* group was 27 years (interquartile range [IQR]: 21–37), while in the control group, it was 34 years (IQR: 28–40). In terms of gender distribution, the *C. trachomatis* group consisted of 32 females (82.05%), whereas the control group had 231 females (49.46%) (Table 2). 

### 3.1. C. trachomatis and HLA-DR and -DQ Alleles

First, we checked the relationships between *HLA-DQB1* and *HLA-DRB1* alleles and *C. trachomatis* (Table 3). We found that the DRB1*13 allele increased the odds of *C. trachomatis* (*p* = 0.017).

Concerning *HLA-DRB4* within the *C. trachomatis* group, we observed four participants with the 01:03:01:02N allele.

### 3.2. C. trachomatis and Genotypes

Next, we checked the relationships between *HLA*-*DRB1* and *HLA*-*DQB1* genotypes and *C. trachomatis* (Table 4). We found that the DQB1*03/DQB1*06 genotype and DRB1*01/DRB1*13 genotype increased the odds of *C. trachomatis.*

After the univariate analyses, we proceeded to assess whether the genotypes that had a statistically significant association with *C. trachomatis* would continue to do so after adjusting for known confounders. Thus, we fit two multiple logistic regression models predicting the presence of *C. trachomatis*, with one genotype as an independent variable of interest, which were adjusted for age and sex (Table 5). After adjustment, the DRB1*01/DRB1*13 genotype retained a statistically significant association with *C. trachomatis*, its presence increasing the odds of the disease. The DQB1*03/DQB1*06 genotype lost its significance after adjustment.

### 3.3. C. trachomatis and Haplotypes

Table 6 presents the description of the *HLA* haplotypes that are most commonly detected in both the *C. trachomatis* and the control groups. We found no associations using the score statistics. 

## 4. Discussion

In our study, we observed a significant increase in the likelihood of *C. trachomatis* infection among individuals carrying the DRB1*13 allele. Subsequently, we investigated the relation between *HLA*-*DRB1* and *HLA*-*DQB1* genotypes and *C. trachomatis* infection. Our analysis revealed that both the DRB1*01/DRB1*13 genotype and DQB1*03/DQB1*06 genotype were associated with elevated odds of *C. trachomatis* infection. However, following adjustment, only the presence of the DRB1*01/DRB1*13 genotype retained statistical significance in association with *C. trachomatis*, indicating a significant increase in disease risk. Conversely, the significance of the DQB1*03/DQB1*06 genotype was lost after adjustment. Furthermore, we performed the examination of the *HLA* haplotypes common in both the *C. trachomatis* and control groups, but it yielded no significant associations using score statistics.

The influence of HLA-DRB1*13 on the increased likelihood of persistent *C. trachomatis* infection has been documented in only one prior study, which focused on Colombian women and specifically highlighted the presence of the HLA-DRB1*13:05:01 allele (*p* = 0.032). Numerous haplotypes associated with *C. trachomatis* occurrence were identified, such as DRB1*01:02:01G-DQB1*03:03:02G or DRB1*12:01:01G-DQB1*03:02:01G, although specific genotypes were not documented [12,18].

The study has several strengths. To our knowledge, this is the first investigation to evaluate HLA alleles, genotypes, or phenotypes associated with *C. trachomatis* infection in the Caucasian population found in Romania. We conducted molecular diagnosis of *C. trachomatis* and compared positive cases with healthy controls from the same exact geographical area of Romania, specifically the counties encompassed in the region of Transylvania. This approach allowed for a comprehensive assessment of the relationship between HLA genetics and *C. trachomatis* susceptibility within a well-defined population.

### 4.1. Study Strengths

Our choice to explore this area of research was motivated by the inconsistency observed in the medical literature regarding which HLA alleles influence the risk of *C. trachomatis* infection, reinfection, recurrence, and complications, as well as those providing protection against the disease. Furthermore, most existing studies have focused on Afro-American populations, leaving a knowledge gap regarding HLA associations in Caucasian populations. Therefore, our study fills an important void by providing insights specific to the Caucasian population and shedding light on the role of HLA in bacterial sexually transmitted diseases.

### 4.2. Limitations

However, there are limitations to consider. The sample size may restrict the generalizability of our findings, and completion of further research with larger cohorts is desirable. We examined a total of 307 individuals who presented symptoms suggestive of bacterial sexually transmitted diseases or were identified as partners of confirmed chlamydia cases. Only 38 individuals, constituting 12.37% of the sample, tested positive for *C. trachomatis*. We attribute the low number of positive cases to the impact of the COVID-19 pandemic, as our study commenced following the outbreak of SARS-CoV-2 and coincided with the implementation of quarantine and isolation measures mandated by authorities during the pandemic. This trend aligns with observations made by the European Centre for Disease Prevention and Control (ECDC), which noted a decline in chlamydia notification rates during 2020 and 2021 amidst the COVID-19 pandemic, followed by a resurgence in 2022 [3].

According to the ECDC, national notification rates for chlamydia infection across Europe varied from 0.1 to 709 cases per 100,000 people. In Romania, the reported chlamydia infection rate in 2021 was merely 0.02 cases per 100,000 individuals, translating to only six reported cases nationwide for the entire year [3]. This scarcity of cases posed a significant challenge in patient recruitment. In Romania, the prevailing stigma associated with STIs often deters individuals from seeking medical attention for such conditions. Additionally, conducting this investigation and identifying chlamydia patients proved to be a formidable challenge. Moreover, despite the existence of a national sexually transmitted disease register, cases from private hospitals are not registered there. It is worth noting that all chlamydia cases are typically tested in private medical centers due to the unavailability of molecular diagnosis through NAAT for this pathology in public hospitals. Discrepancies in chlamydia testing protocols, case identification methods, and reporting practices are believed to exert a greater influence on reported chlamydia figures than actual variations in disease prevalence. We used consecutive sampling in the dermatovenerology, urology, and obstetrics-gynecology departments. But a small proportion of subjects (2%) of the study were recruited using non-consecutive sampling. This implies that the impact of this selection method on the study is minimal. For HLA typing we used sequence-specific primers (SSP-PCR), which works better than sequence-specific oligonucleotide probes (SSO). SSO might have issues in distinguishing between closely related alleles. For the future, an increase in the resolution of HLA typing (also including field 2 regarding specific HLA proteins) might prove beneficial in characterizing more in-depth HLA-pathogen interactions.

Another constraint of the study is the assessment being limited to *HLA-DR* and *HLA-DQ* alleles, neglecting other HLA class I and class II alleles such as *HLA-A*, *HLA-B*, *HLA-C*, and *HLA-DP*. This selection was primarily dictated by financial considerations, prompting us to focus on alleles commonly implicated in this pathology, as reported by previous studies.

To build on our study’s limitations, there are several future research directions. First, expanding our investigation to include a larger sample size and a broader population would enhance the robustness of our findings and allow for a more nuanced analysis of the relationship between HLA alleles and infection susceptibility. Second, exploring additional HLA alleles beyond HLA-DR and HLA-DQ, such as HLA-A, HLA-B, HLA-C, and HLA-DP, as well as increasing the resolution of HLA typing, would provide a more complete picture of the involved genetic factors. Investigating these alleles could reveal new associations and insights into host–pathogen interactions. Third, studying single-nucleotide polymorphisms (SNPs) within these alleles could offer further insights into genetic variations that influence susceptibility to infection and the severity of disease.

Bacteria have developed resistance to multiple drugs and heterotypic resistance. Despite the availability of antibiotics, a vaccine could significantly lower the rates of chlamydial infections [33]. The immune defense against C. trachomatis involves activating CD4+ T cells [34]. An epitope is a segment of an antigen that plays a crucial role in triggering the body’s immune defense. Reverse vaccinology involves using computational genome analysis to predict surface epitopes that are essential for developing potential vaccines [35]. CD4 cells, also known as helper T cells, interact with MHC class II proteins to regulate immune responses by activating other immune cells, such as macrophages and monocytes. T-cell activation and differentiation require three key signals: interaction between the peptide and the HLA molecule, the involvement of cytokines that promote clonal expansion, and signaling through co-stimulatory molecules [36]. Given the discovery of peptides that demonstrate selectivity for both B and T cells, along with extensive population coverage, high conservation, and notable binding interactions with the MHC-1 HLA alleles [35], it is important to also explore the potential of MHC class II molecules. Thus, this investigation, which is missing from the three key signals of T-cell activation, could aid in developing new vaccine models.

In future investigations, exploring HLA alleles influencing the innate immune response and inflammatory pathways may facilitate the creation of an effective vaccine against *C. trachomatis*. Studies have shown the potential of utilizing *HLA-DR4* transgenic mice for testing vaccination antigens relevant to humans. For instance, intranasal immunization with recombinant CPAF was employed to bolster CD4+ T-cell IFN-gamma-dependent protective immunity in *HLA-DR4* transgenic mice [37].

## 5. Conclusions

In conclusion, our study highlights the significant association between specific *HLA* alleles and *C. trachomatis* infection susceptibility in the Caucasian population of Romania. The presence of the *HLA*-DRB1*13 allele was found to significantly increase the likelihood of *C. trachomatis* infection. Additionally, our analysis revealed that the DRB1*01/DRB1*13 genotype remained independently associated with *C. trachomatis* infection even after adjustment, indicating its potential role as a genetic risk factor for the disease. Conversely, while the DQB1*03/DQB1*06 genotype showed an initial association with *C. trachomatis* infection, this significance was lost after adjustment. These findings underscore the importance of genetic factors in shaping individual susceptibility to *C. trachomatis* infection, providing valuable insights for future research and potential personalized healthcare interventions.

## Figures and Tables

**Table 1:** Temperature profile of RT-PCR.

	Temperature °С	Time	Cycle Repeats
Hold	95	15 min	1
Cycling	95	5 s	5
	60	20 s	
	72	15 s	
Cycling 2	95	5 s	40
	60	30 s (fluorescence detection)	
	72	15 s	

**Table 2:** Participants’ characteristics.

Group	*C. trachomatis* (*n* = 38)	Control (*n* = 467)	*p*
Age (year), median (IQR)	27 (21–37)	34 (28–40)	0.002
Sex (f), *n* (%)	32 (82.05)	231 (49.46)	<0.001

**Table 3:** *HLA-DRB1* and *HLA*-*DQB1* alleles’ associations with *C. trachomatis.*

Allele, *n* (%)	*C. trachomatis* (*n* = 38)	Control (*n* = 467)	OR (95% CI), *p*-Value
DRB1*01	8 (10.53)	88 (9.42)	1.13 (95% CI 0.53–2.43), 0.752
DRB1*03	4 (5.26)	115 (12.31)	0.4 (95% CI 0.14–1.1), 0.067
DRB1*04	5 (6.58)	90 (9.64)	0.66 (95% CI 0.26–1.68), 0.38
DRB1*07	12 (15.79)	92 (9.85)	1.72 (95% CI 0.89–3.3), 0.101
DRB1*08	1 (1.32)	12 (1.28)	1.02 (95% CI 0.13–7.99), 1
DRB1*09	0 (0)	4 (0.43)	0 (95% CI 0–NaN), 1
DRB1*10	0 (0)	17 (1.82)	0 (95% CI 0–NaN), 0.632
DRB1*11	15 (19.74)	176 (18.84)	1.06 (95% CI 0.59–1.91), 0.848
DRB1*12	1 (1.32)	11 (1.18)	1.12 (95% CI 0.14–8.78), 0.611
DRB1*13	14 (18.42)	91 (9.74)	2.09 (95% CI 1.13–3.88), 0.017
DRB1*14	3 (3.95)	53 (5.67)	0.68 (95% CI 0.21–2.24), 0.793
DRB1*15	7 (9.21)	107 (11.46)	0.78 (95% CI 0.35–1.75), 0.552
DRB1*16	2 (2.63)	78 (8.35)	0.3 (95% CI 0.07–1.23), 0.076
DQB1*02	9 (11.84)	185 (19.81)	0.54 (95% CI 0.27–1.11), 0.09
DQB1*03	24 (31.58)	319 (34.15)	0.89 (95% CI 0.54–1.47), 0.648
DQB1*04	2 (2.63)	12 (1.28)	2.08 (95% CI 0.46–9.45), 0.284
DQB1*05	15 (19.74)	259 (27.73)	0.64 (95% CI 0.36–1.15), 0.132
DQB1*06	16 (21.05)	159 (17.02)	1.3 (95% CI 0.73–2.32), 0.372

OR, odds ratio; CI, confidence interval; *, separator between gene and allele group.

**Table 4:** *HLA*-*DRB1* and *HLA*-*DQB1* associations with *C. trachomatis.*

Genotype, *n* (%)	*C. trachomatis* (*n* = 38)	Control (*n* = 467)	*p*-Value	Genotype, n (%)	*C. trachomatis* (*n* = 38)	Control (*n* = 467)	*p*-Value
*DQB1 02/02*	1 (2.63)	19 (4.07)	1	*DRB1 04/14*	0 (0)	3 (0.64)	1
*DQB1 02/03*	3 (7.89)	65 (13.92)	0.296	*DRB1 04/15*	0 (0)	4 (0.86)	1
*DQB1 02/04*	1 (2.63)	1 (0.21)	0.145	*DRB1 04/16*	0 (0)	5 (1.07)	1
*DQB1 02/05*	2 (5.26)	55 (11.78)	0.293	*DRB1 07/07*	1 (2.63)	5 (1.07)	0.376
*DQB1 02/06*	0 (0)	26 (5.57)	0.246	*DRB1 07/10*	0 (0)	4 (0.86)	1
*DQB1 03/03*	1 (2.63)	62 (13.28)	0.07	*DRB1 07/11*	2 (5.26)	22 (4.71)	0.7
*DQB1 03/04*	0 (0)	3 (0.64)	1	*DRB1 07/12*	1 (2.63)	1 (0.21)	0.145
*DQB1 03/05*	5 (13.16)	81 (17.34)	0.509	*DRB1 07/13*	2 (5.26)	5 (1.07)	0.091
*DQB1 03/06*	8 (21.05)	46 (9.85)	0.05	*DRB1 07/14*	0 (0)	5 (1.07)	1
*DQB1 04/05*	0 (0)	5 (1.07)	1	*DRB1 07/15*	2 (5.26)	11 (2.36)	0.255
*DQB1 04/06*	1 (2.63)	3 (0.64)	0.269	*DRB1 07/16*	0 (0)	7 (1.5)	1
*DQB1 05/05*	0 (0)	34 (7.28)	0.097	*DRB1 08/11*	0 (0)	2 (0.43)	1
*DQB1 05/06*	6 (15.79)	50 (10.71)	0.416	*DRB1 08/13*	1 (2.63)	3 (0.64)	0.269
*DQB1 06/06*	0 (0)	17 (3.64)	0.63	*DRB1 08/15*	0 (0)	2 (0.43)	1
*DRB1 01/01*	0 (0)	5 (1.07)	1	*DRB1 08/16*	0 (0)	2 (0.43)	1
*DRB1 01/03*	1 (2.63)	14 (3)	1	*DRB1 09/11*	0 (0)	1 (0.21)	1
*DRB1 01/04*	0 (0)	14 (3)	0.614	*DRB1 09/16*	0 (0)	2 (0.43)	1
*DRB1 01/07*	1 (2.63)	6 (1.28)	0.424	*DRB1 10/11*	0 (0)	3 (0.64)	1
*DRB1 01/08*	0 (0)	1 (0.21)	1	*DRB1 10/13*	0 (0)	2 (0.43)	1
*DRB1 01/10*	0 (0)	3 (0.64)	1	*DRB1 10/14*	0 (0)	1 (0.21)	1
*DRB1 01/11*	2 (5.26)	16 (3.43)	0.637	*DRB1 10/15*	0 (0)	1 (0.21)	1
*DRB1 01/13*	4 (10.53)	7 (1.5)	0.006	*DRB1 10/16*	0 (0)	2 (0.43)	1
*DRB1 01/14*	0 (0)	4 (0.86)	1	*DRB1 11/11*	0 (0)	15 (3.21)	0.617
*DRB1 01/15*	0 (0)	10 (2.14)	1	*DRB1 11/12*	0 (0)	1 (0.21)	1
*DRB1 01/16*	0 (0)	3 (0.64)	1	*DRB1 11/13*	2 (5.26)	18 (3.85)	0.656
*DRB1 03/03*	0 (0)	6 (1.28)	1	*DRB1 11/14*	3 (7.89)	10 (2.14)	0.066
*DRB1 03/04*	1 (2.63)	12 (2.57)	1	*DRB1 11/15*	2 (5.26)	17 (3.64)	0.646
*DRB1 03/07*	1 (2.63)	12 (2.57)	1	*DRB1 11/16*	0 (0)	10 (2.14)	1
*DRB1 03/08*	0 (0)	1 (0.21)	1	*DRB1 12/13*	0 (0)	1 (0.21)	1
*DRB1 03/10*	0 (0)	1 (0.21)	1	*DRB1 12/14*	0 (0)	1 (0.21)	1
*DRB1 03/11*	0 (0)	23 (4.93)	0.244	*DRB1 12/15*	0 (0)	1 (0.21)	1
*DRB1 03/12*	0 (0)	2 (0.43)	1	*DRB1 12/16*	0 (0)	2 (0.43)	1
*DRB1 03/13*	1 (2.63)	8 (1.71)	0.508	*DRB1 13/13*	0 (0)	8 (1.71)	1
*DRB1 03/14*	0 (0)	9 (1.93)	1	*DRB1 13/14*	0 (0)	2 (0.43)	1
*DRB1 03/15*	0 (0)	11 (2.36)	1	*DRB1 13/15*	1 (2.63)	8 (1.71)	0.508
*DRB1 03/16*	0 (0)	10 (2.14)	1	*DRB1 13/16*	2 (5.26)	13 (2.78)	0.313
*DRB1 04/04*	0 (0)	4 (0.86)	1	*DRB1 14/14*	0 (0)	2 (0.43)	1
*DRB1 04/07*	1 (2.63)	9 (1.93)	0.546	*DRB1 14/15*	0 (0)	10 (2.14)	1
*DRB1 04/08*	0 (0)	1 (0.21)	1	*DRB1 14/16*	0 (0)	4 (0.86)	1
*DRB1 04/09*	0 (0)	1 (0.21)	1	*DRB1 15/15*	0 (0)	10 (2.14)	1
*DRB1 04/11*	2 (5.26)	23 (4.93)	1	*DRB1 15/16*	0 (0)	12 (2.57)	1
*DRB1 04/12*	0 (0)	2 (0.43)	1	*DRB1 16/16*	0 (0)	3 (0.64)	1
*DRB1 04/13*	1 (2.63)	8 (1.71)	0.508				

**Table 5:** Multivariate logistic regression models predicting the presence of *C. trachomatis*, with specific human leucocyte antigen genotypes as the variable of interest, and adjusted for age and sex.

Variables	OR Adjusted	(95% CI)	*p*
Model 1			
* DRB1 01/13*	14.28	(1.88–296.03)	0.023
* *Age >= 34 years	0.33	(0.14–0.7)	0.005
* *Sex (m vs. f)	0.21	(0.08–0.51)	0.001
Model 2			
* DQB1 03/06*	2.13	(0.78–5.51)	0.127
* *Age >= 34 years	0.34	(0.15–0.71)	0.005
* *Sex (m vs. f)	0.19	(0.07–0.44)	<0.001

OR, odds ratio; CI, confidence interval. The area under the receiver operating characteristic curves for models 1 and 2 was 75.46 (95% CI 68.42–82.51) and 75.55 (95% CI 68.71–82.4), respectively.

**Table 6:** The most frequent *HLA* haplotypes in the *C. trachomatis* group and control group.

*C. trachomatis*			Control		
*DRB1*	*DQB1*	Probability	*DRB1*	*DQB1*	Probability
11	3	0.20044931	11	3	0.18189007
13	6	0.17280171	3	2	0.12312634
1	5	0.12460800	1	5	0.09421840
7	2	0.09766620	4	3	0.08973657
15	6	0.09389683	15	6	0.08454623
4	3	0.06782375	13	6	0.08024561
7	3	0.06511080	16	5	0.07815326
14	5	0.04672800	7	2	0.06506893
3	2	0.04069425	14	5	0.05563832
16	5	0.03115200	7	3	0.03343214

## Data Availability

The raw data supporting the conclusions of this article will be made available by the authors on request.
